# Susceptibility and Response of Human Blood Monocyte Subsets to Primary Dengue Virus Infection

**DOI:** 10.1371/journal.pone.0036435

**Published:** 2012-05-04

**Authors:** Kok Loon Wong, Weiqiang Chen, Thavamalar Balakrishnan, Ying Xiu Toh, Katja Fink, Siew-Cheng Wong

**Affiliations:** Singapore Immunology Network (SIgN), Agency for Science, Technology and Research (ASTAR), Biopolis, Singapore, Singapore; Imperial College London, United Kingdom

## Abstract

Human blood monocytes play a central role in dengue infections and form the majority of virus infected cells in the blood. Human blood monocytes are heterogeneous and divided into CD16^−^ and CD16^+^ subsets. Monocyte subsets play distinct roles during disease, but it is not currently known if monocyte subsets differentially contribute to dengue protection and pathogenesis. Here, we compared the susceptibility and response of the human CD16^−^ and CD16^+^ blood monocyte subsets to primary dengue virus *in vitro*. We found that both monocyte subsets were equally susceptible to dengue virus (DENV2 NGC), and capable of supporting the initial production of new infective virus particles. Both monocyte subsets produced anti-viral factors, including IFN-α, CXCL10 and TRAIL. However, CD16^+^ monocytes were the major producers of inflammatory cytokines and chemokines in response to dengue virus, including IL-1β, TNF-α, IL-6, CCL2, 3 and 4. The susceptibility of both monocyte subsets to infection was increased after IL-4 treatment, but this increase was more profound for the CD16^+^ monocyte subset, particularly at early time points after virus exposure. These findings reveal the differential role that monocyte subsets might play during dengue disease.

## Introduction

The human peripheral blood monocyte population can be divided into two distinct subsets based on CD16 expression [1], [2]. Under healthy physiological conditions, the CD16^−^ monocyte population comprises of approximately 90% of total monocytes, while the remaining 10% consist of CD16^+^ monocytes. CD16^−^ monocytes are predisposed to produce IL-10, while CD16^+^ monocytes produce more pro-inflammatory cytokines, for example TNF-α [3], [4]. CD16^−^ express CCR2 while CD16^+^ monocytes preferentially express CX_3_CR1 [5], and exhibit different movement and migratory behaviors [Bibr pone.0036435-Geissmann1] [Bibr pone.0036435-Auffray1] [Bibr pone.0036435-Ancuta1] [6−9]. Monocyte subsets are known to play different roles during disease conditions. For example, CD16^+^ monocytes are elevated during several inflammatory and infectious conditions, and have been proposed to play pro-inflammatory roles during diseases [10]. The CD16^+^ monocyte subset has also been shown to be more permissive to HIV infection [11].

Dengue virus is a single stranded positive sense RNA virus that belongs to the family *Flaviviridae*. Dengue virus exists as four serotypes, DENV-1 to 4 and is transmitted to humans via the bite of an *Aedes* mosquito [12], [13]. Infection with dengue is asymptomatic in the majority of cases [Bibr pone.0036435-Balmaseda1] [Bibr pone.0036435-Burke1] [Bibr pone.0036435-Endy1] [14−17], but it may also cause dengue fever, a debilitating flu-like illness that lasts for up to two weeks. In rare cases, infection results in dengue hemorrhagic fever or dengue shock syndrome, severe life threatening diseases characterized by high fever with vascular leakage and hemorrhage [18], [19]. The incidence of dengue has risen considerably over the recent decade and it is now a major public health problem [20].

Monocytes are natural host cells for dengue virus [21], [22]. Monocytes have been implicated in both pathogenesis and protection of dengue. Monocytes can produce IFN-α in response to dengue virus [23]. The depletion of monocytes in a murine model of dengue infection resulted in a tenfold increase in systemic viral titers, demonstrating the important role for monocytes in systemic viral control [24]. On the other hand, monocytes promote dengue pathogenesis by being the primary vessels of virus propagation [25], [26]. During secondary immune responses, monocyte infection could be facilitated through antibody dependent enhancement, leading to increased infected cell numbers and higher viral load [27], [28]. Monocytes/macrophages can produce cytokines and chemokines that compromise the integrity of the endothelial cell layer [Bibr pone.0036435-Bosch1] [Bibr pone.0036435-Carr1] [Bibr pone.0036435-Chen1] [Bibr pone.0036435-Chen2] [29−33], possibly leading to vascular leakage, the hallmark of severe dengue disease [18], [27]. Increased numbers of CD16^+^ activated monocytes were found in dengue patients [34].

Evidence suggests that severe manifestations of dengue could be caused by an inappropriate Th2-biased immune response. For example, levels of IL-13 are elevated in patients with dengue hemorrhagic fever [35]. Gene expression profiling of blood cells of children with dengue shock syndrome showed increased transcripts of anti-inflammatory and repair/remodeling genes [36], which happens through the “alternative” activation of monocytes with Th2 cytokines. *In vitro*, pre-treatment of human primary monocytes with Th2 cytokines, including IL-4 and IL-13, results in enhanced susceptibility to dengue virus [37] through the increased expression of dengue virus binding receptors, DC-SIGN (CD209) [38] and mannose receptor (CD206) [37].

Given the vital role of monocytes in dengue virus infection, it is important to delineate how monocyte subsets could differently support dengue virus replication and contribute to the protective and pathogenic manifestations of dengue. In order to investigate the differential roles of monocyte subsets in a primary dengue virus infection setting, we isolated the CD16^+^ and CD16^−^ human blood monocyte subsets from healthy donors and exposed them to dengue virus (DENV2 NGC) *in vitro*. Our results revealed that both monocyte subsets are similarly susceptible to dengue virus infection. The two monocyte subsets were also able to effectively produce protective anti-viral soluble factors. However, monocyte subsets differed in the production of inflammatory soluble factors associated with dengue pathogenesis and inflammation. Under Th2 conditions mimicked by IL-4 treatment, the susceptibility of monocyte subsets was enhanced, but this affected the CD16^+^ monocyte more than the CD16^−^ monocyte subset. The results herein reveal that monocyte subsets can have similar susceptibility and protective mechanisms against dengue, but may fundamentally differ in the mechanisms of dengue pathogenesis.

## Materials and Methods

### Isolation of Human Blood Monocyte Subsets

All blood samples and procedures in this study were approved by the National University of Singapore Institutional Review Board, approval number NUS 1076, in accordance to guidelines of the Health Sciences Authority of Singapore. Informed written consent was taken by the staff of the blood bank of the Health Science Authority of Singapore, in accordance to the Declaration of Helsinki. Peripheral blood mononuclear cells (PBMCs) were obtained from blood of healthy donors using Ficoll-Hypaque (GE Healthcare, Singapore) density gradient centrifugation at 1200×g for 30 mins at room temperature. CD16^−^ and CD16^+^ monocyte subsets were isolated using the CD16 monocyte isolation kit (Miltenyi Biotec, Singapore) according to the manufacturer’s instructions, with some modifications. Briefly, NK cells, neutrophils and T cells were depleted using microbeads conjugated to anti-CD56, anti-CD15 and anti-CD3 antibodies. After depletion, CD16^+^ monocytes were isolated using anti-CD16 conjugated microbeads. CD16^−^ monocytes were subsequently isolated from the negative fraction using anti-CD14 conjugated microbeads. The purity of the isolated monocyte subsets was consistently >90%.

### Infection of Cells with Dengue Virus

Following isolation, monocytes were exposed to DENV2 NGC at a multiplicity of infection (MOI) of 10. After 3 h, monocyte subsets were washed twice and cultured in 96 well U-bottomed plates at 2×10^5^ cells per well in 200 µl of RPMI 1640 medium (Invitrogen, Gibco, Singapore) supplemented with 100 µg/100 U/ml Streptomycin/Penicillin (Sigma-Aldrich) and 10% Fetal bovine serum (FBS) (Gibco). All cells were cultured in a 37°C 5% CO_2_ incubator. For protection assays, K562 cells (ATCC, Manassas, VA**)** were pre-treated for 24 h with supernatants from monocyte subsets with or without dengue virus exposure. Prior to pre-treatment, these supernatants were filtered through 100 kDa centrifuge filters (Millipore, Billerica, MA) at 3000×g, 45 mins at room temperature, to remove virus by size exclusion. Pre-treated K562 cells were washed and infected with DENV2 NGC at a MOI of 2. After 1 h, cells were washed and cultured in RPMI 1640 media with 10% FBS. After 2 days, the extent of infection was determined by flow cytometry using anti-NS1 (Non-structural protein 1) intracellular labeling. Data were collected on a BD FACS calibur (BD Bioscience, CA, San Jose).

### Propagation of Dengue Virus

DENV 2 NGC was used for this study. For virus propagation, C6/36 cells were initially exposed to the virus at MOI of 0.01. After 1 h, the cells were washed and cultured at 28°C in RPMI 1640 supplemented with 5% FBS. At days 4, 5 and 6, cell supernatants were harvested. Cell debris was removed by centrifugation at 1000×g for 10 mins and stored at −80°C. Virus titers were determined by plaque assay.

### Plaque Assay

BHK-21 (ATCC, Manassas, VA**)** cells were seeded into 24 well plates and grown to confluence. Serially diluted supernatants containing dengue virus were added to the cells and incubated for 1 h at 37°C in a 5% CO_2_ incubator. Cells were then overlaid with RPMI medium containing 0.4% carboxymethylcellulose (Merck Calbiochem) and incubated for 4 1/2 days at 37°C in a 5% CO_2_ incubator. The cells were then fixed with 1% formalin before plaques were revealed by staining with 1% crystal violet (Merck Calbiochem). Virus titres are thus expressed as plaque forming units per milliliter (PFU/ml).

### Cytokine and Chemokine Detection

The concentrations of cytokines and chemokines reported in this study were determined using multiplex bead arrays kits (Bio-Plex Pro Human Cytokine 27 and 21 plex kits) (Biorad, Hercules, CA) according to the manufacturer’s instructions. Plates were read using a Luminex 200 (Qiagen, Valencia, CA). IFN-α levels were determined using IFN-α multi-subtype ELISA kit according to the manufacturer’s instructions (PBL Interferon Source, Piscataway, NJ). Absorbance at OD450 nm was measured by a Tecan M200 microplate reader (Männedorf, Switzerland).

### Detection of Intracellular Antigens by Flow Cytometry

Cells were fixed with PBS containing 4% paraformaldehyde (Sigma-Aldrich) for 20 min at room temperature, before permeabilization with PBS containing 0.5% BSA and 0.1% saponin (Sigma-Aldrich). Cells were labeled with anti-NS1 PE (a kind gift of Dr. Dennis Burton, The Scripps Research Institute, La Jolla, USA) and anti-E-protein (Envelope protein) (4G2) allophycocyanin (ATCC, Manassas, VA) for 45 mins at room temperature. Following washing with permeabilization buffer the cells were analyzed using a FACS Calibur (BD Bioscience).

### Viability Assays

For Annexin V and 7-AAD (7-Aminoactinomycin D) staining, cells were washed and resuspended in Annexin V buffer containing 140 nM NaCl, 2.5 mM CaCl_2_ and 10 mM Hepes/NaOH. Cells were subsequently stained with Annexin-V PE (BD Bioscience) and 7-AAD (eBioscience, San Diego, CA) for 15 min and analysed by flow cytometry. Live and dead cells were also discriminated using a Live/Dead® cell stain kit (Invitrogen) comprising a far red reactive dye with an emission of 660 nm. Live cells actively exclude this dye and stain dimly, while dead cells are unable to exclude the dye and stain brightly. Staining was performed in PBS according to the manufacturer’s protocol, before analysis by flow cytometry. A MTS assay which detects the dehydrogenase activity of metabolically active cells was performed using CellTiter 96® AQ_ueous_ kit (Promega, Singapore) according to the manufacturer’s protocol. Dehydrogenase activity converts MTS into a formazan product was measured by its absorbance at OD490 nm using a Tecan M200 microplate reader. Absorbance at OD490 nm is directly proportional to the number of viable cells in culture.

### IL-4 Pretreatment of Monocyte Subsets

Isolated monocyte subsets were seeded into flat-bottom 6-well plates at a cell density of 1×10^6^ cells/ml. Monocyte subsets were treated with recombinant human IL-4 (Immunotools) at concentration of 25 ng/ml. As controls, monocyte subsets were cultured without IL-4 treatment. The cells were incubated at 37°C, 5% CO2 for 2 days. The monocyte subsets were washed with culture media prior to exposure to dengue virus at a MOI of 10.

### Statistical Analysis

The statistical significance of the results was determined by a two-tailed Student’s *t* test. Differences with *p*<0.05 were considered significant.

## Results

### CD16^−^ and CD16^+^ Monocyte Populations are Equally Susceptible to Dengue Virus Infection

**Figure 1:**
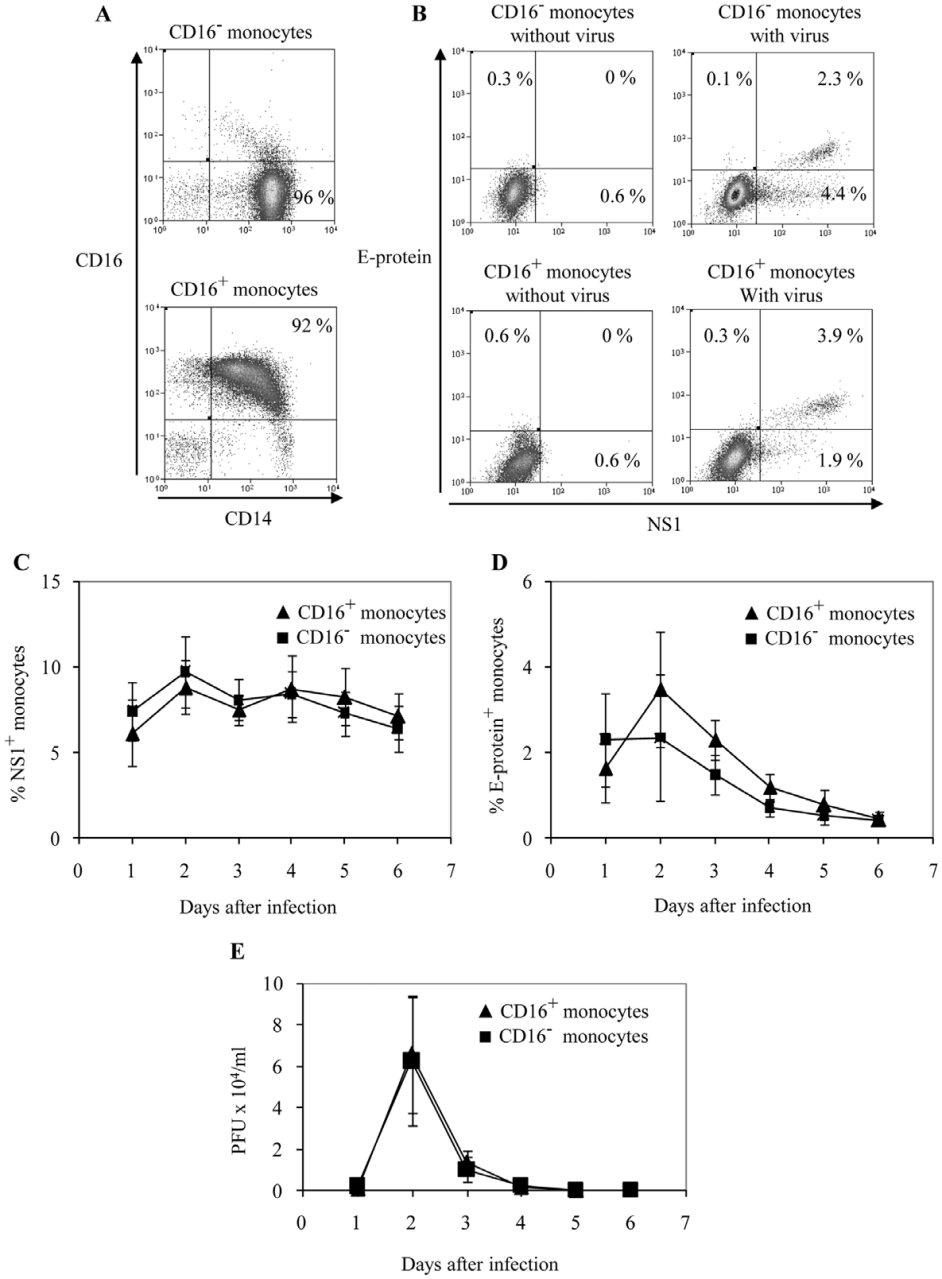
Susceptibility of monocyte subsets to dengue virus infection. (A) Flow cytometric profile of CD16^−^ and CD16^+^ monocytes after isolation. (B) Isolated CD16^−^ or CD16^+^ monocyte subsets were either exposed to dengue virus (DENV2, NGC) at a MOI of 10 or medium without virus. After 2 days, monocytes were fixed, permeabilized and labeled with anti-E-protein and anti-NS1 specific antibodies. Quadrants for virus exposed monocytes (right panel) were set based on monocytes without virus exposure (left panel). Percentage positive cells in each quadrant are shown. Representative data for 6 different donors. (C and D) Percentages of CD16^−^ and CD16^+^ monocytes that are NS1^+^ or E-protein^+^ over the course of 6 days after virus exposure. Results are mean ± SE of 6 different donors. (E) Plaque assays with BHK-21 cells were performed with supernatants taken from virus exposed CD16^−^ or CD16^+^ monocytes over the course of 6 days. Results are mean ± SE from 5 different donors.

We first compared the susceptibility of CD16^−^ and CD16^+^ monocyte subsets to dengue virus infection. CD16^−^ and CD16^+^ monocytes were isolated from PBMCs of healthy blood donors. Typical purities of the isolated monocyte subsets are shown in Fig. 1 A. Freshly isolated monocyte subsets were subsequently exposed to dengue virus (DENV2, NGC strain) at a MOI of 10. A relatively high MOI of 10 was chosen because it consistently gave results that we detectable by intracellular staining. Infected cells were identified by intracellular labeling with antibodies specific for NS1 and E-protein of dengue virus. NS1 is a non-structural protein that is required for virus replication, whereas E-protein is a structural protein that is required to package RNA into new virus particles to be released from infected cells [39]. Using intracellular flow cytometry analysis, NS1 and E-protein were detected in monocytes that were exposed to dengue virus but not controls without virus (Fig 1 B). NS1 and E-protein expression was followed over a course of six days (Fig. 1 C & D). The percentages of NS1 or E-protein positive cells were similar for both CD16^+^ and CD16^−^ monocytes after virus exposure. These results show that both monocyte subsets were equally susceptible to dengue virus infection.

We next determined whether both monocyte subsets had the ability to support production of infectious virus particles. The supernatants of virus exposed monocyte subsets were harvested over the course of six days and tested for the presence of newly produced infective virus by plaque assay on BHK-21 cells (Fig. 1 E). At day 1 post infection, relatively low amounts of infective virus were detected. Subsequently, the amount of virus produced increased dramatically, which peaked at day 2. This then rapidly declined to almost undetectable levels by day 5. Both monocyte subsets supported the production of infective virus to a similar extent and with similar kinetics.

### Soluble Factors Associated with Protection Against Dengue are Produced by Both Monocyte Subsets

**Figure 2:**
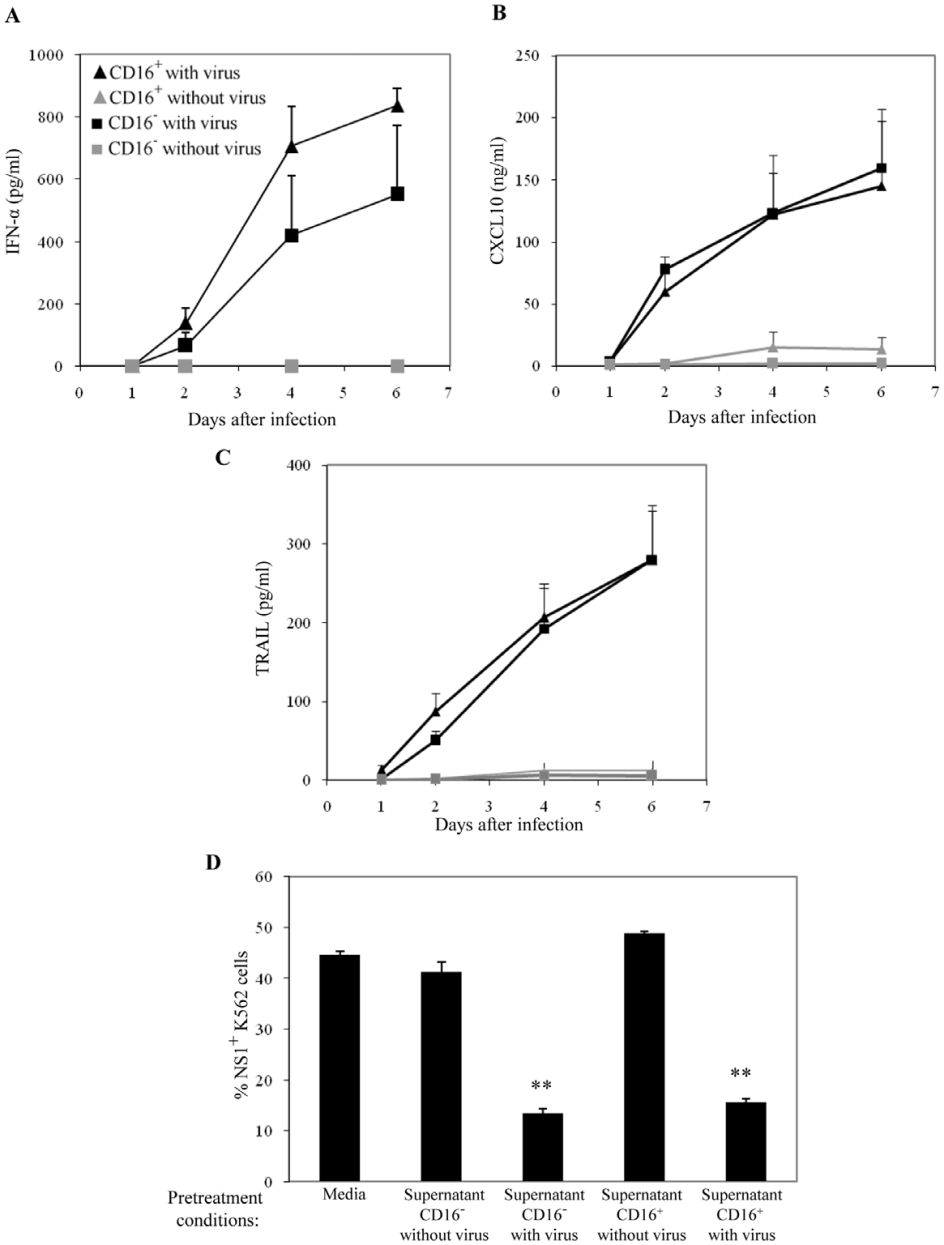
Production of soluble factors associated with protection against dengue by monocyte subsets. Isolated monocyte subsets were either exposed to dengue virus (DENV2, NGC) at a MOI of 10 or medium without virus. Supernatants were harvested over the course of 6 days. (A) Levels of IFN-α were determined by a multi-subtype specific ELISA kit. (B and C) Levels of CXCL10 and TRAIL were determined using multiplex bead arrays. Results are mean ± SE for 6 different donors. There were no significant differences were found between infected CD16^−^ and CD16^+^ monocytes. (D) Supernatants from CD16^−^ and CD16^+^ monocytes exposed to dengue virus or medium without virus were harvested at day 6. These supernatants were passed through 100 kDa centrifuge filters to remove dengue virus. K562 cells were pretreated for 24 hours with either culture medium, supernatants of CD16^−^ or CD16^+^ monocytes with or without virus exposure. Pre-treated K562 cells were washed and infected with dengue virus at a MOI of 2. After 2 days, the extent of infection was determined by intracellular labeling of K562 cells with anti-NS1 antibody. The percentage of NS1^+^ K562 cells after 2 days is shown. Data are representative of 2 experiments using different donors. **, *p*<0.005 between respective monocyte subset with and without virus.

Cytokines and chemokines produced by monocytes play vital roles in dengue pathogenesis and protection [Bibr pone.0036435-Bosch1] [Bibr pone.0036435-Carr1] [Bibr pone.0036435-Chen1] [Bibr pone.0036435-Chen2] [29−33]. We were interested to understand if cytokines and chemokines that play a key role in dengue were differentially produced by monocyte subsets. Using multiplex bead arrays and ELISAs, we observed that cytokines and chemokines associated with protection against dengue, namely CXCL10 [40], [41], IFN-α [42], [43] and TRAIL [44] are produced by both monocyte subsets in response to dengue virus (Fig. 2 A, B, C). Interestingly, the production of these three soluble factors increased steadily over the course of 6 days in both monocyte subsets. These factors were present at much higher levels after the peak of new infective virus production at day 2, however there was no significant difference between the levels of these factors in CD16^+^ and CD16^−^ virus exposed monocytes. Hence, both monocyte subsets were equally capable of producing protective factors against dengue virus, although the major part of this response occurred late (>4 days) after exposure to virus.

Next, we determined the ability of virus exposed monocyte subsets to confer protection against dengue virus infection. Supernatants of monocyte subsets 6 days after infection were used, since they contained the highest levels of factors associated with protection against dengue. Residual virus in these supernatants was first removed by size exclusion using 100 kDa centrifuge filters. This efficiently reduces infective virus loads by 10^5^ fold, but retains the levels of soluble factors by >80% (data not shown). For infection, we used K562 cells which are highly susceptible to infection by dengue virus [43]. K562 cells were pre-treated overnight with filtered supernatants from day 6 virus exposed monocyte subsets, non-exposed monocyte subset control supernatants or medium only. Pre-treated K562 cells were subsequently washed and exposed to dengue virus at a MOI of 2. After 2 days, the levels of intracellular NS1 were determined (Fig. 2 D). We observed that the percentage of NS1 positive K562 was three fold lower in cells that had been pre-treated with supernatants of virus exposed monocytes, as compared to K562 cells pre-treated with control monocyte supernatants or medium only. This showed that the supernatants of virus exposed monocytes could significantly reduce the extent of dengue virus infection of K562 cells. When comparing CD16^−^ and CD16^+^ monocytes, no significant difference in the reduction of K562 infection was observed. Hence, monocyte subsets infected with dengue virus produced protective factors, including IFN-alpha and IP-10. Hence, we speculate that these protective factors present in supernatants used to treat K562 cells protected them from infection by dengue virus.

### Inflammatory Cytokines and Chemokines are Predominantly Produced by CD16^+^ Monocytes

**Figure 3:**
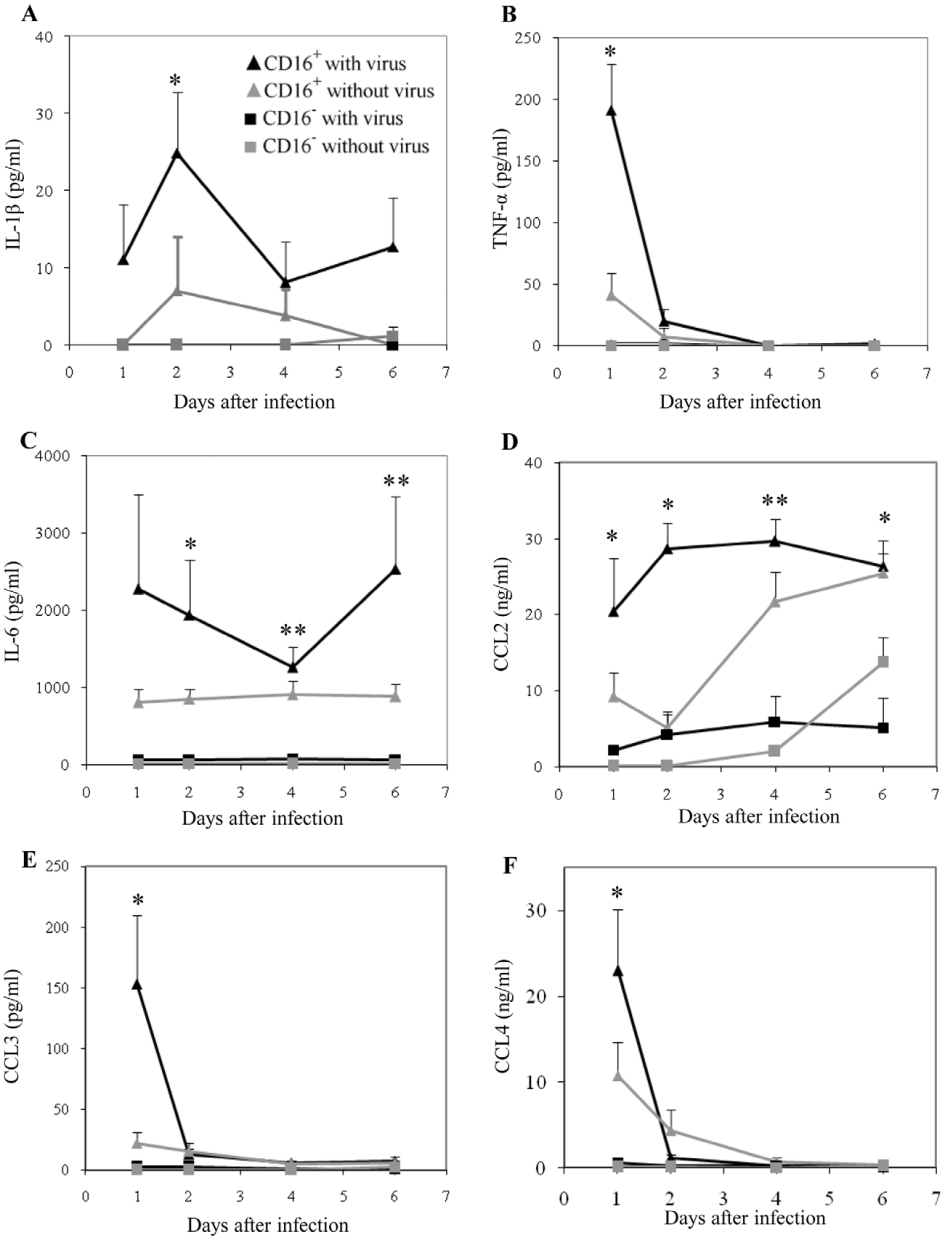
Production of inflammatory cytokines by monocyte subsets. Monocyte subsets were exposed to dengue virus or medium without virus. Supernatants were harvested over the course of 6 days. Levels of (A) IL-1β (B) TNF-α (C) IL-6 (D) CCL2 (E) CCL3 and (F) CCL4 were measured using multiplex bead arrays. Results are mean ± SE of 5 different donors. * *p*<0.05, **, *p*<0.005 between CD16^+^ and CD16^−^ monocytes with virus.

In contrast to the soluble factors associated with dengue protection, differences were observed with the production of inflammatory cytokines. The levels of IL-1β, IL-6 and TNF-α produced by CD16^+^ monocytes were significantly higher than that of CD16^−^ monocytes in response to dengue virus. Virus exposed CD16^+^ monocytes produced picogram levels of IL-1β and TNF-α, but these cytokines were undetectable with CD16^−^ monocytes (Fig. 3 A & B). Furthermore, the level of IL-6 produced by CD16^+^ monocytes was more than 30 times higher than the levels observed with CD16^−^ monocytes at day 2 (1939.9±717.2 versus 58.1±21.0) (Fig. 3 C). Hence, CD16^+^ monocytes are the major producers of inflammatory cytokines in response to dengue virus.

The production of CC chemokines, in particular CCL2 (MCP-1), CCL3 (MIP-1α) and CCL4 (MIP-1β), was also clearly different between CD16^+^ and CD16^−^ monocytes. The levels of CCL2, 3 and 4 were significantly higher in supernatants of virus exposed CD16^+^ monocytes compared to virus exposed CD16^−^ monocytes. The level of CCL2 was more than five fold higher for virus exposed CD16^+^ versus CD16^−^ monocytes at any time tested (Fig. 3 D). While the level of CCL3 from virus exposed CD16^+^ monocytes was 153.1±56.7 pg/ml at day 1, it was barely detectable in CD16^−^ monocyte cultures (Fig. 3 E). Similarly, CCL4 production was 40 fold higher in CD16^+^ monocytes than CD16^−^ monocytes at day 1 (22950±7221 pg/ml vs 570±195) (Fig. 3 F). The production of CCL3 and CCL4 by virus exposed CD16^+^ monocytes was transient, and rapidly declined after day 1. However, the levels of CCL2 remained relatively constant over 6 days after virus exposure.

### Viability of Both Monocyte Subsets is Enhanced after Exposure to Dengue Virus

**Figure 4:**
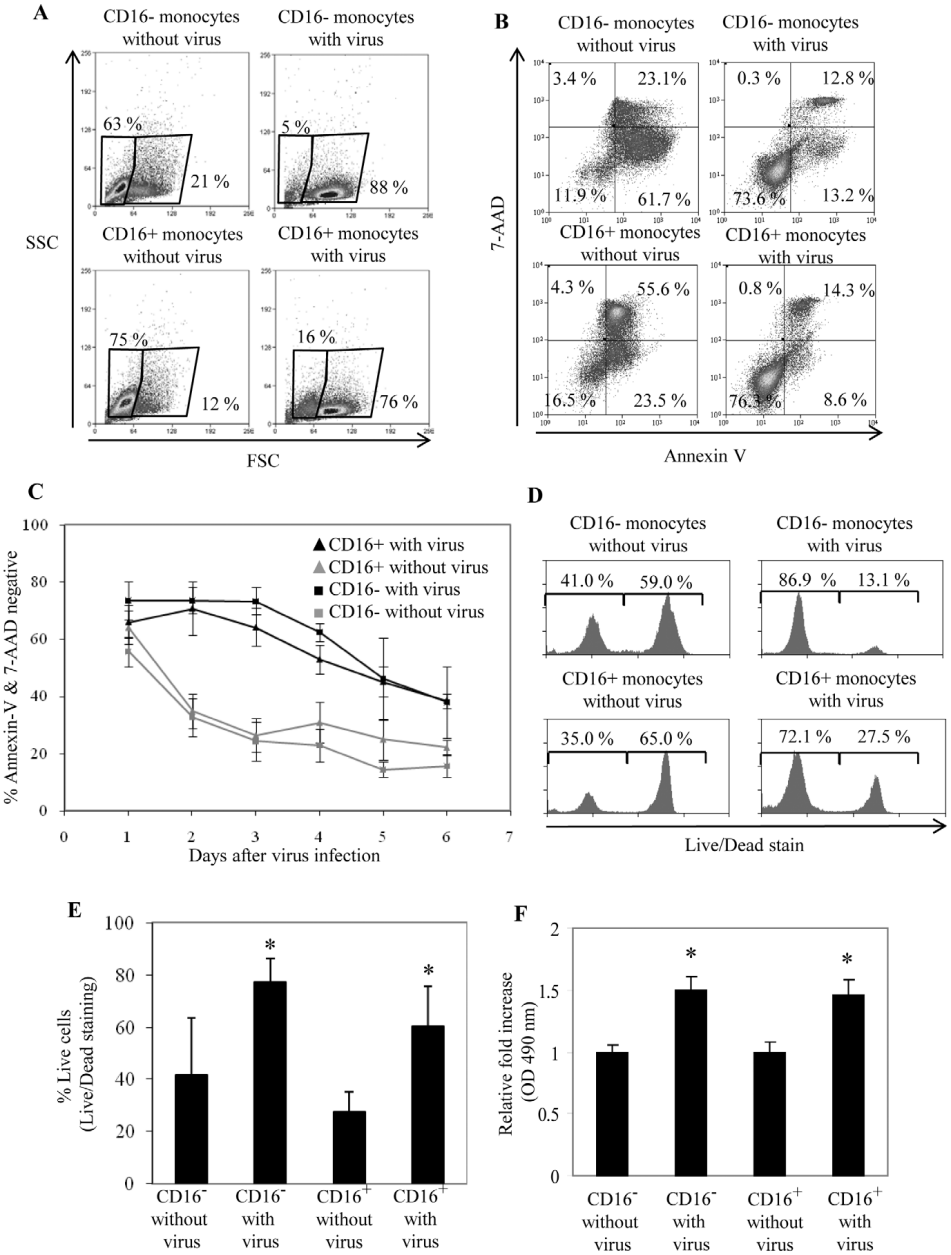
Viability of monocyte subsets after dengue virus exposure. (A) Representative forward scatter (FSC) versus side scatter (SSC) plot of monocyte subsets exposed to dengue virus or medium without virus after 2 days. Numbers indicate percentages within each region gate. (B) Annexin V and 7-AAD staining of monocyte subsets exposed to dengue virus or medium without virus after 2 days. Representative data for 5 different donors. (C) Percentage of viable monocyte subsets (Annexin V and 7-AAD double negative) over the course of 6 days after exposure to dengue virus or medium without virus. Data are expressed as mean ± SE from 5 different donors. (D) Live/Dead staining of monocyte subsets exposed to dengue virus or medium without virus after 2 days. Dead cells stain strongly with the dye (right peaks) while live cells are able to actively exclude the dye and are thus stained weakly (left peaks). Representative data for 4 different donors. (E) Percentage of live cells exposed to dengue virus or medium without virus after 2 days. Data are expressed as mean ± SE from 4 different donors. (F) MTS assay. Metabolic activity of live cells produces a substrate that absorbs at OD490 nm. Absorbance at OD490 nm is proportional to the number of live cells. Data are normalized to the respective monocyte subset without virus. Representative data for 2 different donors. *, *p*<0.05 between respective monocyte subset with and without virus.

Through flow cytometry analysis, we noted differences between the scatter profiles of monocytes that were exposed to dengue virus compared to monocytes without virus. For example at day 2, monocyte subsets without virus consist of a higher percentage of cells with a lower forward scatter and higher side scatter (Fig. 4 A), a profile typical of dead cells. In contrast, dengue virus exposed monocytes contained a greater percentage of cells with higher forward scatter and lower side scatter, which is typical of live cells. This was observed for both the CD16^−^ and CD16^+^ monocytes. To determine whether the viability of monocyte subsets was indeed increased upon virus exposure, we stained the cells with Annexin V and 7-AAD over the course of 6 days (Fig. 4 B & C). There were significantly more viable cells (AnnexinV^-^7-AAD^-^) for both monocyte subsets following exposure to virus compared to monocytes without virus. Fig. 4 B shows a representative dot plot of unexposed and virus exposed monocyte subsets at day 2. Fig. 4 C represents accumulated data for AnnexinV^-^ 7-AAD^-^ cells over the course of 6 days. We also used a Live/Dead cell stain kit that consists of a dye that emits at 660 nm to determine viability. Live cells actively exclude this dye and stain dimly, while dead cells are unable to exclude the dye and stain brightly. The percentages of viable (dimly stained) and dead cells (brightly stained) in virus exposed or monocyte subsets without virus were determined by flow cytometry (Fig. 4 D, E). The results showed that the percentage of viable cells after virus exposure was significantly higher (∼2 fold) for both monocyte subsets. Finally we used a non-flow cytometry based approach to test cell viability. The MTS assay utilizes the dehydrogenase activity of viable cells to convert MTS into a substrate that absorbs at OD490 nm. Absorbance at OD490 nm is proportional to the number of viable cells in culture. For each monocyte subset, the absorbance for virus exposed cells was normalized to monocytes without virus. The results at day 2 showed that absorbance was approximately 1.5 times higher in monocyte cultures with virus (Fig. 4 F). These data show that the viability of both monocyte subsets was similarly enhanced by exposure to dengue virus. Hence, exposure of monocytes to dengue virus could have down-regulated spontaneous apoptosis that occurs normally in non-stimulated monocytes [Bibr pone.0036435-Fahy1] [Bibr pone.0036435-Mangan1] [45−47]. The increased viability of monocyte subsets through dengue virus exposure may serve to enhance the functions of monocytes during dengue virus infection, for example through sustaining their ability to produce cytokines.

### IL-4 Pretreatment of Monocyte Subsets Enhances the Susceptibility of CD16^+^ Monocyte Subsets to a Greater Extent

**Figure 5:**
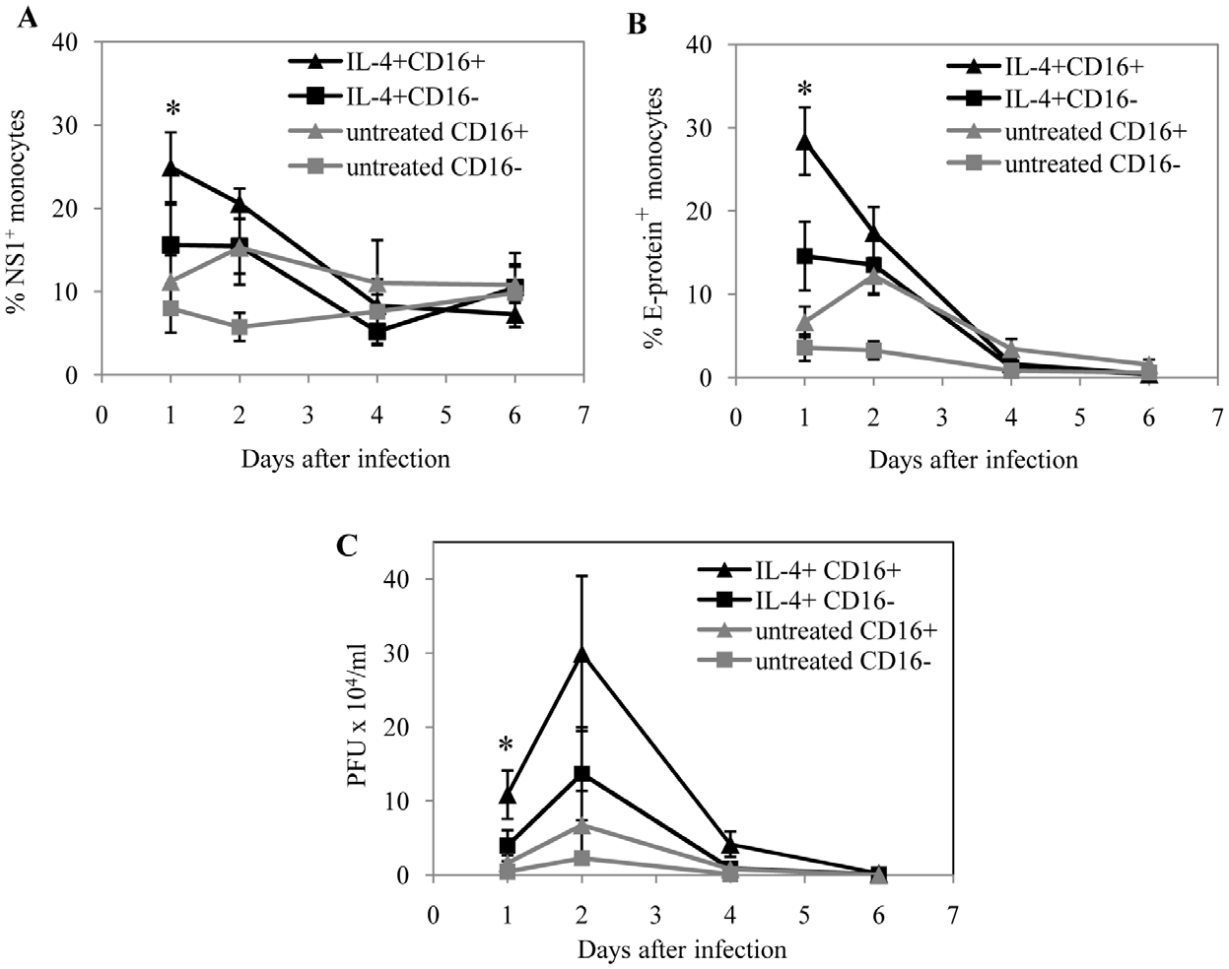
IL-4 treatment enhances the susceptibility of the CD16^+^ monocyte subset to a greater extent. Isolated CD16^−^ or CD16^+^ monocyte subsets were pretreated with 25 ng/ml of IL-4 for two days. Cells were subsequently washed and harvested before exposure to dengue virus (DENV2, NGC) at a MOI of 10 or medium without virus. Percentages of CD16^−^ and CD16^+^ monocytes that are (A) NS1^+^ or (B) E-protein^+^ over the course of 6 days after virus exposure. Results are mean ± SE of 5 different donors. (C) Plaque assays with BHK-21 cells were performed with supernatants taken from virus exposed IL-4 treated CD16^−^ or CD16^+^ monocytes over the course of 6 days. Results are mean ± SE from 4 different donors. * *p*<0.05, between IL-4 treated CD16^+^ and IL-4 treated CD16^−^ monocytes with virus.

It has been previously shown that pre-treatment of human monocytes with IL-4 or IL-13, increases their susceptibility to dengue virus infection *in vitro* [37]. We further investigated if IL-4 treatment of the two monocyte subsets differentially affects their susceptibility to dengue virus infection. For this, CD16^+^ and CD16^−^ monocyte subsets were cultured with IL-4 for two days before exposure to dengue virus. The extent of infection was measured using intracellular staining for NS1 and 4G2 over the course of 6 days after initial exposure (Fig. 5 A & B). IL-4 treatment of both monocyte subsets results in significantly increased percentage of infected cells, as compared to the respective monocyte subset control without IL-4 treatment. However, we observed particularly for day 1 after infection, the extent of NS1 and 4G2 percentage positive cells was significantly greater for IL-4 treated CD16^+^ monocytes as compared to CD16- monocytes. This result suggests that CD16^+^ monocytes became more susceptible than CD16^−^ monocytes after IL-4 treatment. The preferential susceptibility of the CD16^+^ monocytes after IL-4 treatment was also supported by the results of the plague assay, which show that the supernatants of the IL-4 treated CD16^+^ monocyte subset harbored a higher titer of dengue virus compared to the IL-4 CD16^−^ treated monocyte subset (Fig. 5 C).

## Discussion

Monocytes play a central role in dengue infection, but the relative role of monocyte subsets during dengue infection is unclear. We find that freshly isolated healthy monocyte subsets were equally susceptible to dengue virus infection, indicating that at least during normal physiological conditions, dengue virus infection is not preferentially biased towards either subset. We also show that monocyte subsets were equally capable of producing anti-viral factors associated with dengue protection, such as IFN-α, CXCL10 and TRAIL. This demonstrates that both monocyte subsets can potentially contribute to anti-dengue responses through multiple mechanisms. Type 1 interferons like IFN-α are potent anti-virals that limit the propagation of dengue virus [42], [43]. CXCL10 competes with dengue virus for binding to cell surface heparan sulfate, thereby reducing viral uptake and infection of cells [40], [41]. Interestingly, CXCL10 is the ligand for CXCR3, expressed by NK cells and T helper type 1 cells [48], [49]. Though speculative, the production of CXCL10 by monocytes could also be a mechanism for the efficient recruitment and activation of these cells during dengue infection, thereby positively influencing disease outcome in two ways. TRAIL has been identified as a protective factor against dengue infection [44] via a mechanism linked to type 1 interferon responses [50].

CD16^+^ monocytes are known for their preferential ability to produce inflammatory cytokines after stimulation [51], [52]. Consistent with this, CD16^+^ monocytes dominated the production of inflammatory cytokines in response to dengue virus infection. Unregulated production of inflammatory cytokines could contribute to the immune pathogenesis of dengue, such as vascular leakage, which is a hallmark of severe forms of the disease [18], [27]. For example, TNF-α can mediate vascular leakage by increasing the permeability of endothelial cell monolayers [53], [54]. Furthermore, higher serum levels of inflammatory cytokines are associated with severe dengue [Bibr pone.0036435-FernandezMestre1] [Bibr pone.0036435-Hober1] [55−57]. Hence, it is possible that CD16^+^ monocytes contribute to immune pathogenesis through inflammatory mechanisms. However, the production of TNF-α and IL-1β was early and transient in our experiments. The severe dengue symptoms like vascular leakage, which usually occur in the later stages of infection, could be the consequence of continuous differentiation or release of CD16^+^ monocytes into the circulation.

CCL chemokines, in particular CCL2, 3 and 4 were preferentially produced by CD16^+^ monocytes during the early phases of the dengue response in our assays. This may be involved in the recruitment of other immune cells by CD16^+^ monocytes, which could be a protective mechanism to accumulate other immune cells in lymphatic tissue or infected organs during dengue infection. CCL2 is potentially involved in mediating vascular permeability. *In vitro*, CCL2 disrupts tight junctions between endothelial cells and enhances vascular permeability [58]. CCL2 is also present at higher levels in the serum of dengue hemorrhagic fever patients compared to dengue fever patients [58]. The levels of CCL2 produced by CD16^+^ monocytes were relatively constant over time, and might represent a possible mechanism by which CD16^+^ monocytes could mediate vascular leakage in the later phases of the dengue infection. In contrast to CCL2, CCL4 levels have been associated with a good prognosis in dengue infection [59], perhaps through an association with numbers of circulating CD56^+^ NK cells.

Monocytes are known to die spontaneously by apoptosis and this can be prevented with appropriate stimuli [Bibr pone.0036435-Fahy2] [Bibr pone.0036435-Mangan3] [60−62]. Our study showed that the exposure of monocyte subsets to dengue virus prevented the spontaneous apoptosis that occurred in non-stimulated monocytes. Increased viability of infected cells could have important implications *in vivo*, resulting in prolonged cytokine and chemokine production. Interestingly, it has been found that for epithelial cells, flavivirus NS4A protein induces autophagy and this subsequently protects cells from cell death [63]. However, enhanced viability and prolonged survival of infected monocytes needs to be verified in dengue patients.

Th2 cytokines have been known to enhance the susceptibility of primary human monocytes [37]. Here, we extend these results by showing that IL-4 pre-treatment affects the susceptibility of the CD16^+^ monocyte subset to a greater extent. These results may imply that the CD16^+^ monocyte subset may have a bigger contribution to dengue virus pathogenesis during Th2-biased immune responses.

While there are concerns over whether positive selection could have non-specifically activated or perturb the behavior of the monocyte subsets, a microarray study comparing positive and negative selected peripheral blood mononuclear cell (PBMCs) populations reveal that positive selection did not affect cellular transcription in any significant manner [64]. Negative selection was deemed inferior due to lower cell purities which resulted in significant changes to the transcriptome. While there is also a concern that the 10% contaminating cells could have contributed to the results of the study, our preliminary results and those of Kou et al [26] shows that monocytes and not other PBMCs were infected by dengue virus (Fig S1A & B). Furthermore, as CD16 was found to be spontaneously upregulated during culture even without virus (Fig S1C), it was not possible to analyze the infection of monocyte subpopulations without isolating them first. Nevertheless, after infection of whole PBMCs for two days, a small population of CD14+CD16- monocytes could still be observed. By gating on these CD14+CD16- and CD14+CD16+ monocytes, we could see that the infection rates were similar (figure S1D, E & F). Hence, this further supports the notion that prior isolation of monocyte subsets does not affect their susceptibility to dengue virus infection. Furthermore, we found little NS1 and 4G2 expression by CD14- non-monocytes. Hence, the contaminating 10% of non-monocytes in PBMCs are unlikely to contribute to the results obtained in this study. The average percentage of NS1 and 4G2 and standard deviation for the various populations analysed for all three donors are also shown in Fig 1F.

There might also be concerns on whether the results obtained from the lab adapted NGC strain is best suited for infecting primary cells. However, other groups have also successfully infected human monocytes using the 16681 strain, and have found comparable results between the 16681 and NGC strain [26], [65], this suggests that the results obtained for NGC can also be applicable to a broader range of DENV-2 virus strains.

Overall, our results delineate how monocyte subsets can have both similar and different responses to dengue virus infection. This demonstrates the advantage of taking monocyte heterogeneity into account for studies of viral infections like dengue. More recently, monocytes have been further subdivided into three subsets [Bibr pone.0036435-Wong1] [Bibr pone.0036435-Zawada1] [66−68]. Future studies on the individual roles of these subsets may reveal further insights into the contribution of monocyte subsets to viral diseases like dengue. For example, it was shown that amongst the CD16^+^ monocyte subset, the CD14^high^ intermediate subset was selectively expanded in mild but not severe dengue [65]. Furthermore, monocytes are known to be involved in secondary infections that result in antibody dependent enhancement and severe dengue disease. It will be of great interest to determine the relative contribition of monocyte subsets to secondary dengue virus infections. We believe that these endeavors would allow the identification and development of more precise therapeutic strategies for dengue.

## Supporting Information

Figure S1 **Infection of whole PBMCs.** Whole 1 PBMCs were were exposed to dengue virus or medium without virus. After two days, surface staining of CD14 and CD16, and intracellular staining for NS1 and 4G2 was performed. (A) NS1 and 4G2 expression by gating on the whole CD14^+^ monocyte population. (B) NS1 and 4G2 expression by gating on the whole CD14^−^ non monocyte population. (C) CD14 and CD16 profiles demonstrating the spontaneous upregulation of CD16 by monocytes after two days of culture. (D) NS1 and 4G2 expression by gating on the CD14^+^CD16^+^ monocyte population (E) NS1 and 4G2 expression by gating on the CD14^+^CD16 monocyte population. (F) Percentage of NS1^+^ and 4G2 positive cells from the mean + SD of three donors.
(PDF)

## References

[1] Ziegler Heitbrock HW (1996). Heterogeneity of human blood monocytes: the CD14+ CD16+ subpopulation. Immunol.. Today.

[2] Passlick B, Flieger D, Ziegler Heitbrock HW (1989). Identification and characterization of a novel monocyte subpopulation in human peripheral blood.. Blood.

[3] Frankenberger M, Sternsdorf T, Pechumer H, Pforte A, Ziegler Heitbrock HW (1996). Differential cytokine expression in human blood monocyte subpopulations: a polymerase chain reaction analysis.. Blood.

[4] Belge KU, Dayyani F, Horelt A, Siedlar M, Frankenberger M (2002). The proinflammatory CD14+CD16+DR++ monocytes are a major source of TNF. J. Immunol..

[5] Weber C, Belge KU, von HP, Draude G, Steppich B (2000). Differential chemokine receptor expression and function in human monocyte subpopulations. J. Leukoc. Biol..

[pone.0036435-Geissmann1] Geissmann F, Jung S, Littman DR (2003). Blood monocytes consist of two principal subsets with distinct migratory properties. Immunity..

[pone.0036435-Auffray1] Auffray C, Fogg D, Garfa M, Elain G, Join Lambert O (2007). Monitoring of blood vessels and tissues by a population of monocytes with patrolling behavior.. Science.

[pone.0036435-Ancuta1] Ancuta P, Rao R, Moses A, Mehle A, Shaw SK (2003). Fractalkine preferentially mediates arrest and migration of CD16+ monocytes. J. Exp. Med..

[9] Cros J, Cagnard N, Woollard K, Patey N, Zhang SY (2010). Human CD14dim monocytes patrol and sense nucleic acids and viruses via TLR7 and TLR8 receptors. Immunity..

[10] Ziegler Heitbrock L (2007). The CD14+ CD16+ blood monocytes: their role in infection and inflammation. J. Leukoc. Biol..

[11] Ellery PJ, Tippett E, Chiu YL, Paukovics G, Cameron PU (2007). The CD16+ monocyte subset is more permissive to infection and preferentially harbors HIV-1 in vivo. J. Immunol..

[12] Mackenzie JS, Gubler DJ, Petersen LR (2004). Emerging flaviviruses: the spread and resurgence of Japanese encephalitis, West Nile and dengue viruses. Nat. Med..

[13] Henchal EA, Putnak JR (1990). The dengue viruses. Clin. Microbiol. Rev..

[pone.0036435-Balmaseda1] Balmaseda A, Hammond SN, Tellez Y, Imhoff L, Rodriguez Y (2006). High seroprevalence of antibodies against dengue virus in a prospective study of schoolchildren in Managua, Nicaragua. Trop. Med. Int.. Health.

[pone.0036435-Burke1] Burke DS, Nisalak A, Johnson DE, Scott RM (1988). A prospective study of dengue infections in Bangkok. Am. J. Trop. Med. Hyg..

[pone.0036435-Endy1] Endy TP, Chunsuttiwat S, Nisalak A, Libraty DH, Green S (2002). Epidemiology of inapparent and symptomatic acute dengue virus infection: a prospective study of primary school children in Kamphaeng Phet, Thailand. Am. J. Epidemiol..

[17] Thein S, Aung MM, Shwe TN, Aye M, Zaw A (1997). Risk factors in dengue shock syndrome. Am. J. Trop. Med. Hyg..

[18] Halstead SB (2007). Dengue.. Lancet.

[19] Gubler DJ (1998). Dengue and dengue hemorrhagic fever. Clin. Microbiol. Rev..

[20] Kyle JL, Harris E (2008). Global spread and persistence of dengue. Annu. Rev. Microbiol..

[21] Durbin AP, Vargas MJ, Wanionek K, Hammond SN, Gordon A (2008). Phenotyping of peripheral blood mononuclear cells during acute dengue illness demonstrates infection and increased activation of monocytes in severe cases compared to classic dengue fever.. Virology.

[22] Jessie K, Fong MY, Devi S, Lam SK, Wong KT (2004). Localization of dengue virus in naturally infected human tissues, by immunohistochemistry and in situ hybridization. J. Infect. Dis..

[23] Kurane I, Ennis FA (1988). Production of interferon alpha by dengue virus-infected human monocytes. J. Gen. Virol.. 69 (Pt.

[24] Fink K, Ng C, Nkenfou C, Vasudevan SG, van RN (2009). Depletion of macrophages in mice results in higher dengue virus titers and highlights the role of macrophages for virus control. Eur. J. Immunol..

[25] Halstead SB, O’Rourke EJ, Allison AC (1977). Dengue viruses and mononuclear phagocytes. II. Identity of blood and tissue leukocytes supporting in vitro infection. J. Exp. Med..

[26] Kou Z, Quinn M, Chen H, Rodrigo WW, Rose RC (2008). Monocytes, but not T or B cells, are the principal target cells for dengue virus (DV) infection among human peripheral blood mononuclear cells. J. Med. Virol..

[27] Halstead SB (1988). Pathogenesis of dengue: challenges to molecular biology.. Science.

[28] Kliks SC, Nisalak A, Brandt WE, Wahl L, Burke DS (1989). Antibody-dependent enhancement of dengue virus growth in human monocytes as a risk factor for dengue hemorrhagic fever. Am. J. Trop. Med. Hyg..

[pone.0036435-Bosch1] Bosch I, Xhaja K, Estevez L, Raines G, Melichar H (2002). Increased production of interleukin-8 in primary human monocytes and in human epithelial and endothelial cell lines after dengue virus challenge. J. Virol..

[pone.0036435-Carr1] Carr JM, Hocking H, Bunting K, Wright PJ, Davidson A (2003). Supernatants from dengue virus type-2 infected macrophages induce permeability changes in endothelial cell monolayers. J. Med. Virol..

[pone.0036435-Chen1] Chen ST, Lin YL, Huang MT, Wu MF, Cheng SC (2008). CLEC5A is critical for dengue-virus-induced lethal disease.. Nature.

[pone.0036435-Chen2] Chen YC, Wang SY (2002). Activation of terminally differentiated human monocytes/macrophages by dengue virus: productive infection, hierarchical production of innate cytokines and chemokines, and the synergistic effect of lipopolysaccharide. J. Virol..

[33] Spain Santana TA, Marglin S, Ennis FA, Rothman AL (2001). MIP-1 alpha and MIP-1 beta induction by dengue virus. J. Med. Virol..

[34] Azeredo EL, Neves-Souza PC, Alvarenga AR, Reis SR, Torrentes-Carvalho A (2010). Differential regulation of toll-like receptor-2, toll-like receptor-4, CD16 and human leucocyte antigen-DR on peripheral blood monocytes during mild and severe dengue fever.. Immunology.

[35] Mustafa AS, Elbishbishi EA, Agarwal R, Chaturvedi UC (2001). Elevated levels of interleukin-13 and IL-18 in patients with dengue hemorrhagic fever. FEMS Immunol. Med. Microbiol..

[36] Devignot S, Sapet C, Duong V, Bergon A, Rihet P (2010). Genome-wide expression profiling deciphers host responses altered during dengue shock syndrome and reveals the role of innate immunity in severe dengue. PLoS. One..

[37] Miller JL, de Wet BJ, Martinez-Pomares L, Radcliffe CM, Dwek RA (2008). The mannose receptor mediates dengue virus infection of macrophages. PLoS. Pathog..

[38] Tassaneetrithep B, Burgess TH, Granelli Piperno A, Trumpfheller C, Finke J (2003). DC-SIGN (CD209) mediates dengue virus infection of human dendritic cells. J. Exp. Med..

[39] Mukhopadhyay S, Kuhn RJ, Rossmann MG (2005). A structural perspective of the flavivirus life cycle. Nat. Rev. Microbiol..

[40] Chen JP, Lu HL, Lai SL, Campanella GS, Sung JM (2006). Dengue virus induces expression of CXC chemokine ligand 10/IFN-gamma-inducible protein 10, which competitively inhibits viral binding to cell surface heparan sulfate. J. Immunol..

[41] Ip PP, Liao F (2010). Resistance to dengue virus infection in mice is potentiated by CXCL10 and is independent of CXCL10-mediated leukocyte recruitment. J. Immunol..

[42] Stark GR, Kerr IM, Williams BR, Silverman RH, Schreiber RD (1998). How cells respond to interferons. Annu. Rev. Biochem..

[43] Diamond MS, Roberts TG, Edgil D, Lu B, Ernst J (2000). Modulation of Dengue virus infection in human cells by alpha, beta, and gamma interferons. J. Virol..

[44] Warke RV, Martin KJ, Giaya K, Shaw SK, Rothman AL (2008). TRAIL is a novel antiviral protein against dengue virus. J. Virol..

[pone.0036435-Fahy1] Fahy RJ, Doseff AI, Wewers MD (1999). Spontaneous human monocyte apoptosis utilizes a caspase-3-dependent pathway that is blocked by endotoxin and is independent of caspase-1. J. Immunol..

[pone.0036435-Mangan1] Mangan DF, Welch GR, Wahl SM (1991). Lipopolysaccharide, tumor necrosis factor-alpha, and IL-1 beta prevent programmed cell death (apoptosis) in human peripheral blood monocytes. J. Immunol..

[47] Mangan DF, Wahl SM (1991). Differential regulation of human monocyte programmed cell death (apoptosis) by chemotactic factors and pro-inflammatory cytokines. J. Immunol..

[48] Campbell JJ, Qin S, Unutmaz D, Soler D, Murphy KE (2001). Unique subpopulations of CD56+ NK and NK-T peripheral blood lymphocytes identified by chemokine receptor expression repertoire. J. Immunol..

[49] Qin S, Rottman JB, Myers P, Kassam N, Weinblatt M (1998). The chemokine receptors CXCR3 and CCR5 mark subsets of T cells associated with certain inflammatory reactions. J. Clin.. Invest.

[50] Kumar Sinha C, Varambally S, Sreekumar A, Chinnaiyan AM (2002). Molecular cross-talk between the TRAIL and interferon signaling pathways. J. Biol. Chem..

[51] Belge KU, Dayyani F, Horelt A, Siedlar M, Frankenberger M (2002). The proinflammatory CD14+CD16+DR++ monocytes are a major source of TNF. J. Immunol..

[52] Frankenberger M, Sternsdorf T, Pechumer H, Pforte A, Ziegler Heitbrock HW (1996). Differential cytokine expression in human blood monocyte subpopulations: a polymerase chain reaction analysis.. Blood.

[53] Dewi BE, Takasaki T, Kurane I (2004). In vitro assessment of human endothelial cell permeability: effects of inflammatory cytokines and dengue virus infection. J. Virol.. Methods.

[54] Yen YT, Chen HC, Lin YD, Shieh CC, Wu Hsieh BA (2008). Enhancement by tumor necrosis factor alpha of dengue virus-induced endothelial cell production of reactive nitrogen and oxygen species is key to hemorrhage development. J. Virol..

[pone.0036435-FernandezMestre1] Fernandez Mestre MT, Gendzekhadze K, Rivas Vetencourt P, Layrisse Z (2004). TNF-alpha-308A allele, a possible severity risk factor of hemorrhagic manifestation in dengue fever patients.. Tissue Antigens.

[pone.0036435-Hober1] Hober D, Poli L, Roblin B, Gestas P, Chungue E (1993). Serum levels of tumor necrosis factor-alpha (TNF-alpha), interleukin-6 (IL-6), and interleukin-1 beta (IL-1 beta) in dengue-infected patients. Am. J. Trop. Med. Hyg..

[57] Wang L, Chen RF, Liu JW, Yu HR, Kuo HC (2007). Implications of dynamic changes among tumor necrosis factor-alpha (TNF-alpha), membrane TNF receptor, and soluble TNF receptor levels in regard to the severity of dengue infection. Am. J. Trop. Med. Hyg..

[58] Lee YR, Liu MT, Lei HY, Liu CC, Wu JM (2006). MCP-1, a highly expressed chemokine in dengue haemorrhagic fever/dengue shock syndrome patients, may cause permeability change, possibly through reduced tight junctions of vascular endothelium cells. J. Gen. Virol..

[59] Bozza FA, Cruz OG, Zagne SM, Azeredo EL, Nogueira RM (2008). Multiplex cytokine profile from dengue patients: MIP-1beta and IFN-gamma as predictive factors for severity. BMC. Infect. Dis..

[pone.0036435-Fahy2] Fahy RJ, Doseff AI, Wewers MD (1999). Spontaneous human monocyte apoptosis utilizes a caspase-3-dependent pathway that is blocked by endotoxin and is independent of caspase-1. J. Immunol..

[pone.0036435-Mangan3] Mangan DF, Wahl SM (1991). Differential regulation of human monocyte programmed cell death (apoptosis) by chemotactic factors and pro-inflammatory cytokines. J. Immunol..

[62] Mangan DF, Welch GR, Wahl SM (1991). Lipopolysaccharide, tumor necrosis factor-alpha, and IL-1 beta prevent programmed cell death (apoptosis) in human peripheral blood monocytes. J. Immunol..

[63] McLean JE, Wudzinska A, Datan E, Quaglino D, Zakeri Z (2011). Flavivirus NS4A-induced Autophagy Protects Cells against Death and Enhances Virus Replication. J. Biol. Chem..

[64] Lyons PA, Koukoulaki M, Hatton A, Doggett K, Woffendin HB (2007). Microarray analysis of human leucocyte subsets: the advantages of positive selection and rapid purification. BMC.. Genomics.

[65] Azeredo EL, Neves-Souza PC, Alvarenga AR, Reis SR, Torrentes Carvalho A (2010). Differential regulation of toll-like receptor-2, toll-like receptor-4, CD16 and human leucocyte antigen-DR on peripheral blood monocytes during mild and severe dengue fever.. Immunology.

[pone.0036435-Wong1] Wong KL, Tai JJ, Wong WC, Han H, Sem X (2011). Gene expression profiling reveals the defining features of the classical, intermediate, and nonclassical human monocyte subsets.. Blood.

[pone.0036435-Zawada1] Zawada AM, Rogacev KS, Rotter B, Winter P, Marell RR (2011). SuperSAGE evidence for CD14++CD16+ monocytes as a third monocyte subset..

[68] Ziegler Heitbrock L, Ancuta P, Crowe S, Dalod M, Grau V (2010). Nomenclature of monocytes and dendritic cells in blood.. Blood.

