# Supplementary material for: Susceptibility and Response of Human Blood Monocyte Subsets to Primary Dengue Virus Infection
Source: PLoS One. 2012 May 4;7(5):e36435. doi: 10.1371/journal.pone.0036435 (PMC3344872; doi:10.1371/journal.pone.0036435)

Supplementary figure 1A. NS1 and 4G2 expression by whole CD14+ monocytes after 2 days with or without virus infection

Left panels:
PBMCs without virus

Right panels:
PBMCs with virus (MOI 10)

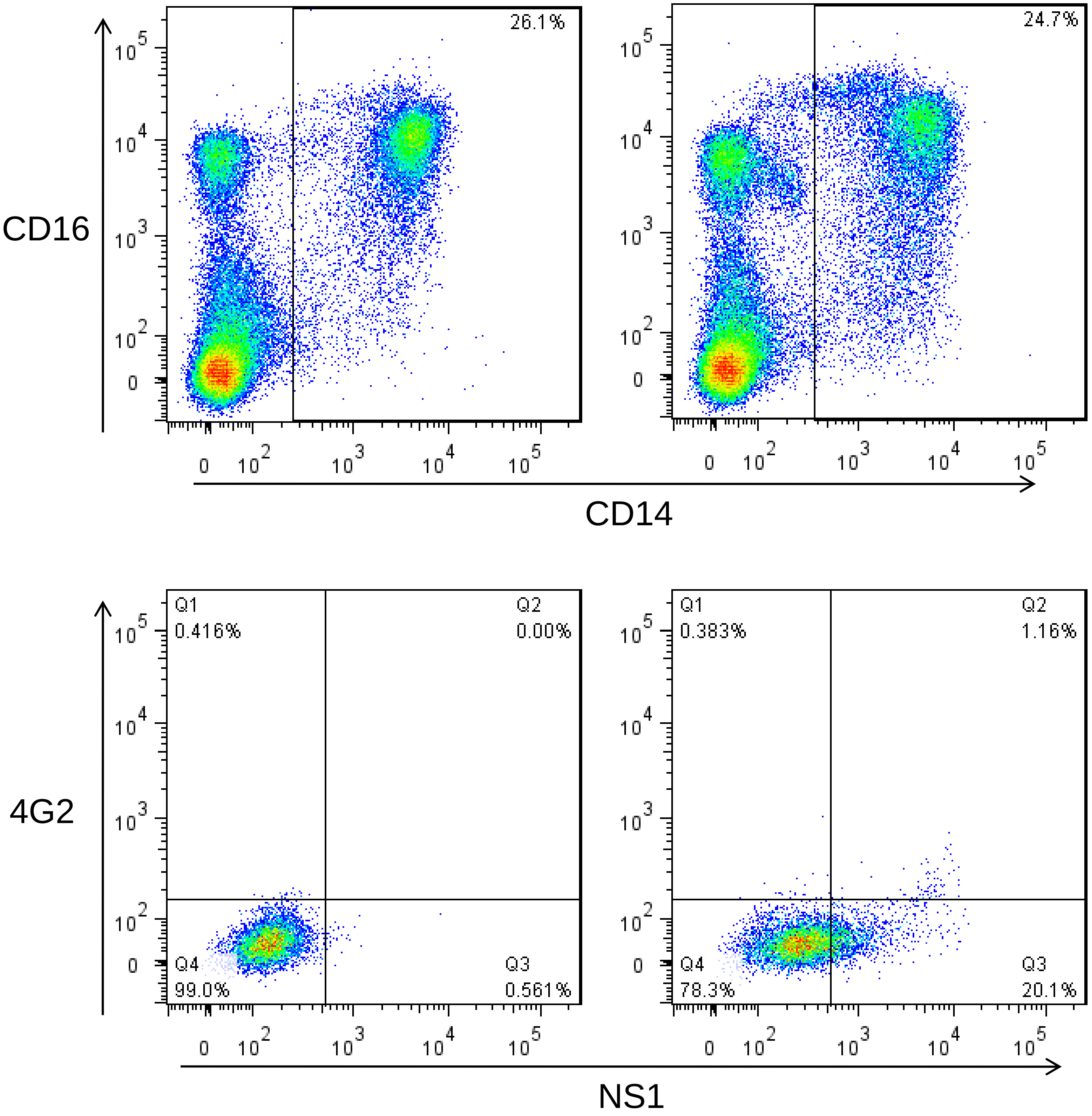

Supplementary figure 1B. NS1 and 4G2 expression by whole CD14- non- monocytes after 2 days with or without virus infection

Left panels:
PBMCs without virus

Right panels:
PBMCs with virus (MOI 10)

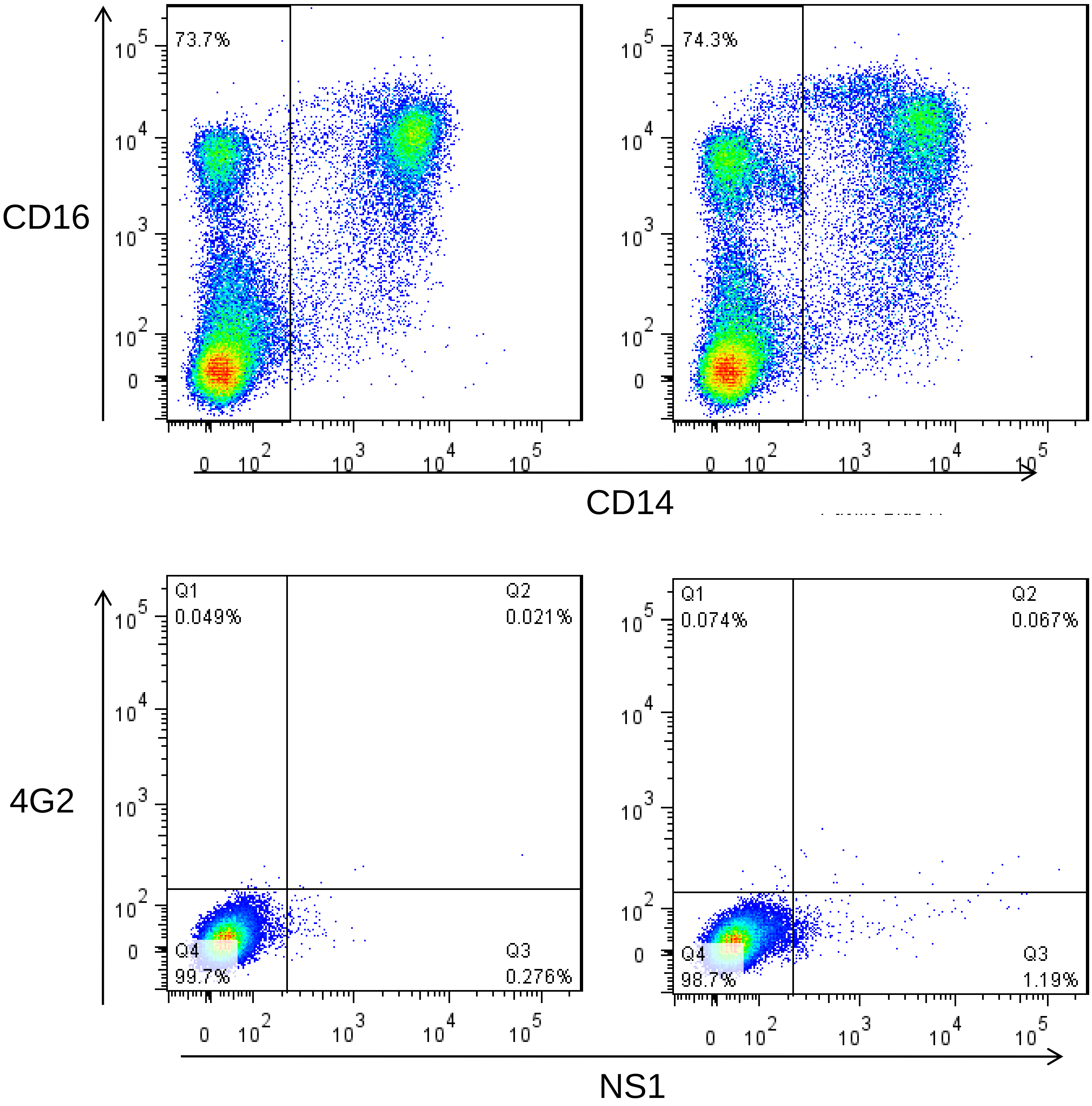

Supplementary figure 1C. CD16- monocytes spontaneously unregulate CD16 expression after culture for 2 days with or without virus

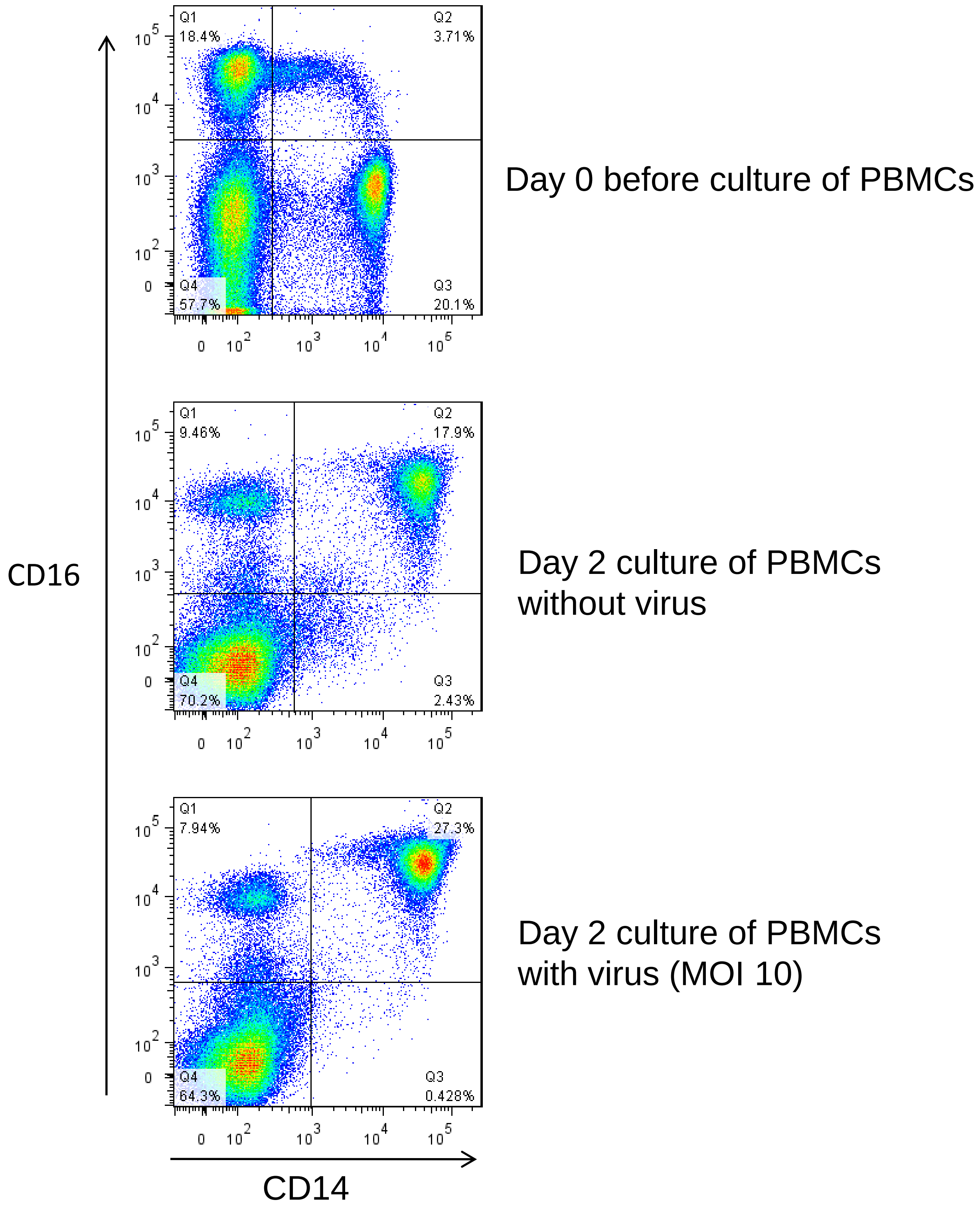

Supplementary figure 1D. NS1 and 4G2 expression by CD14+CD16+ monocytes after 2 days with or without virus infection

Left panels:
PBMCs without virus

Right panels:
PBMCs with virus (MOI 10)

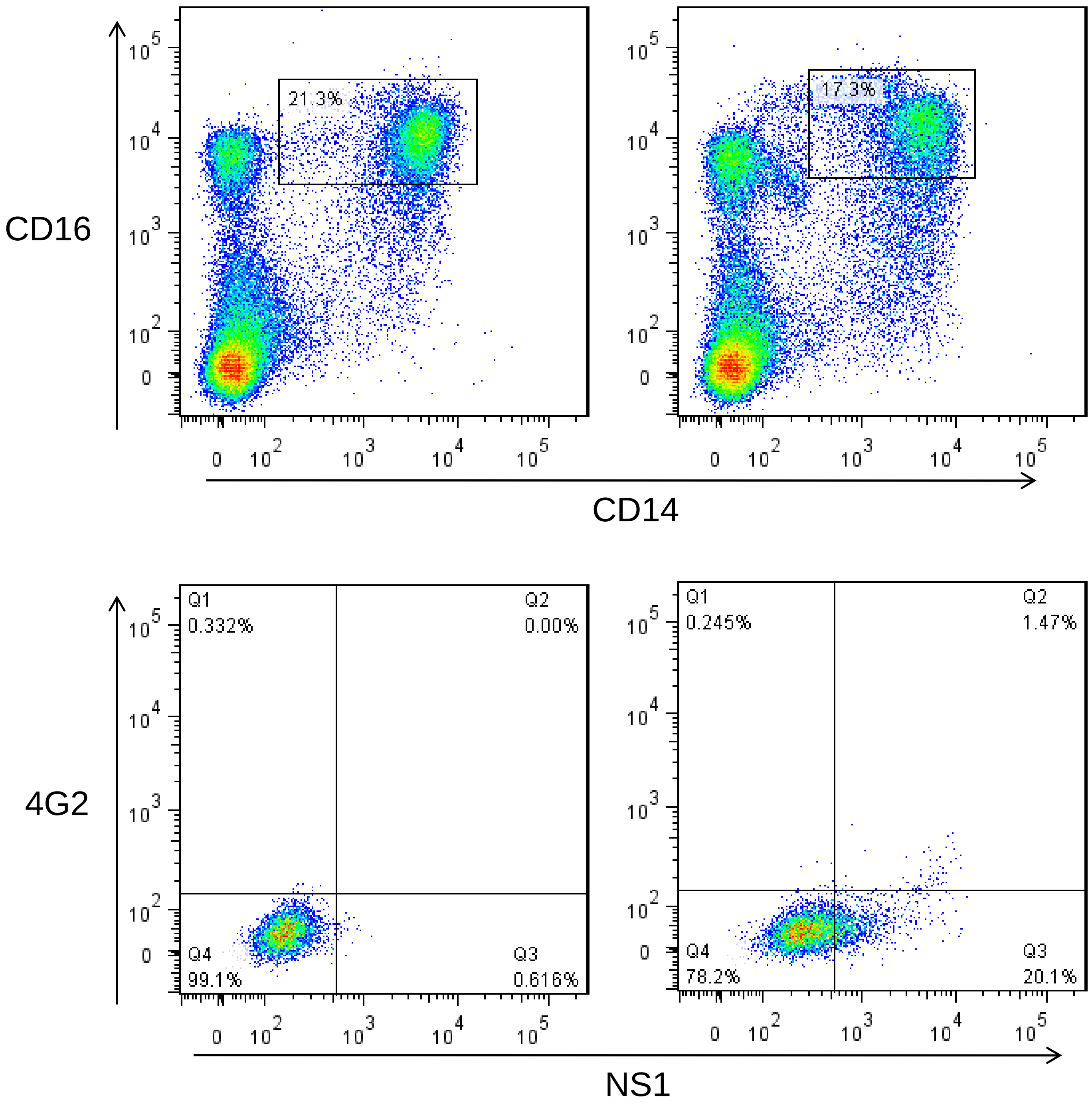

Supplementary figure 1E. NS1 and 4G2 expression by CD14+CD16- monocytes after 2 days with or without virus infection

Left panels:
PBMCs without virus

Right panels:
PBMCs with virus (MOI 10)

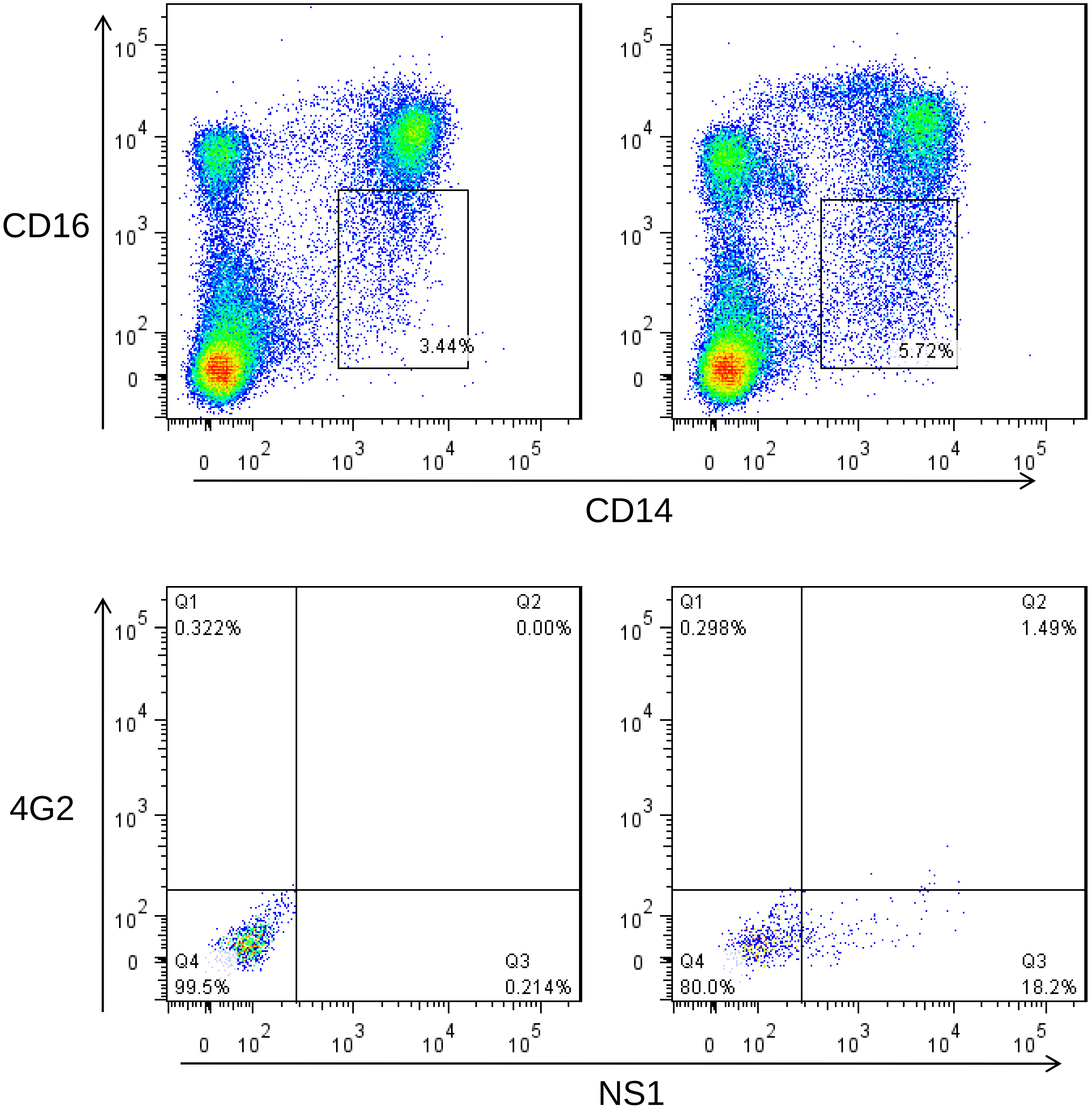

Supplementary figure 1F. NS1 and 4G2 expression by different PBMC populations 2 days after virus exposure. Data are mean $\pm$ SD of three independent donors.

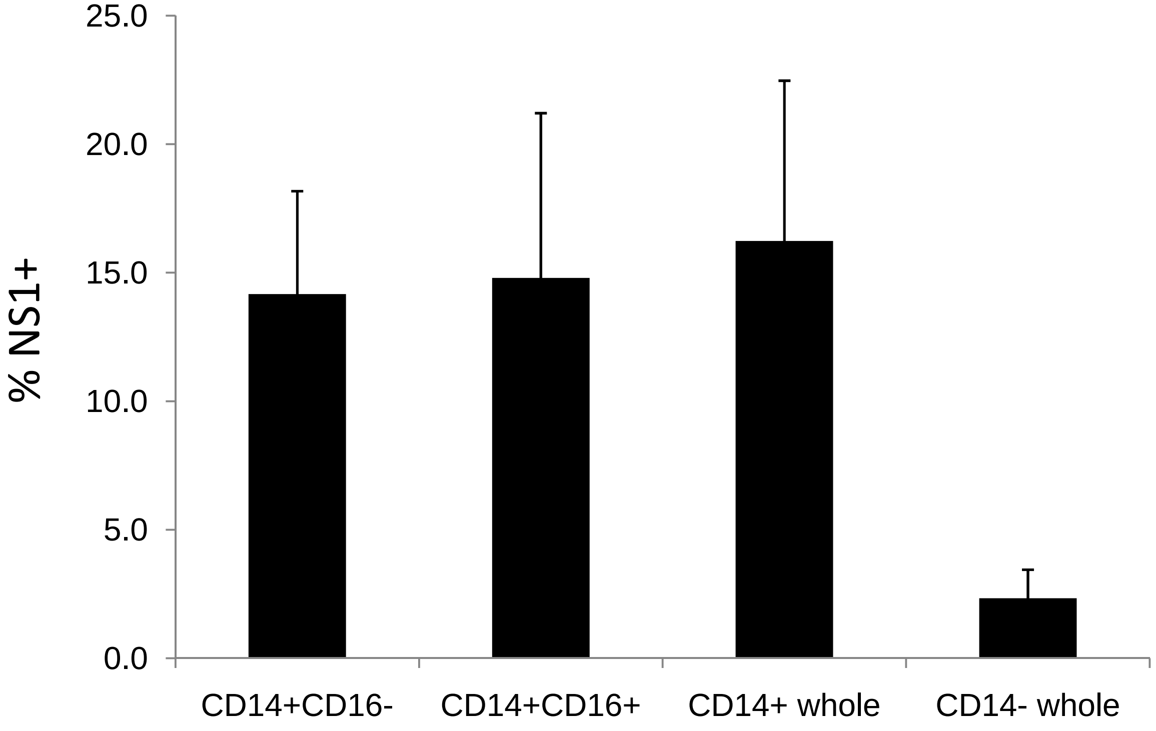

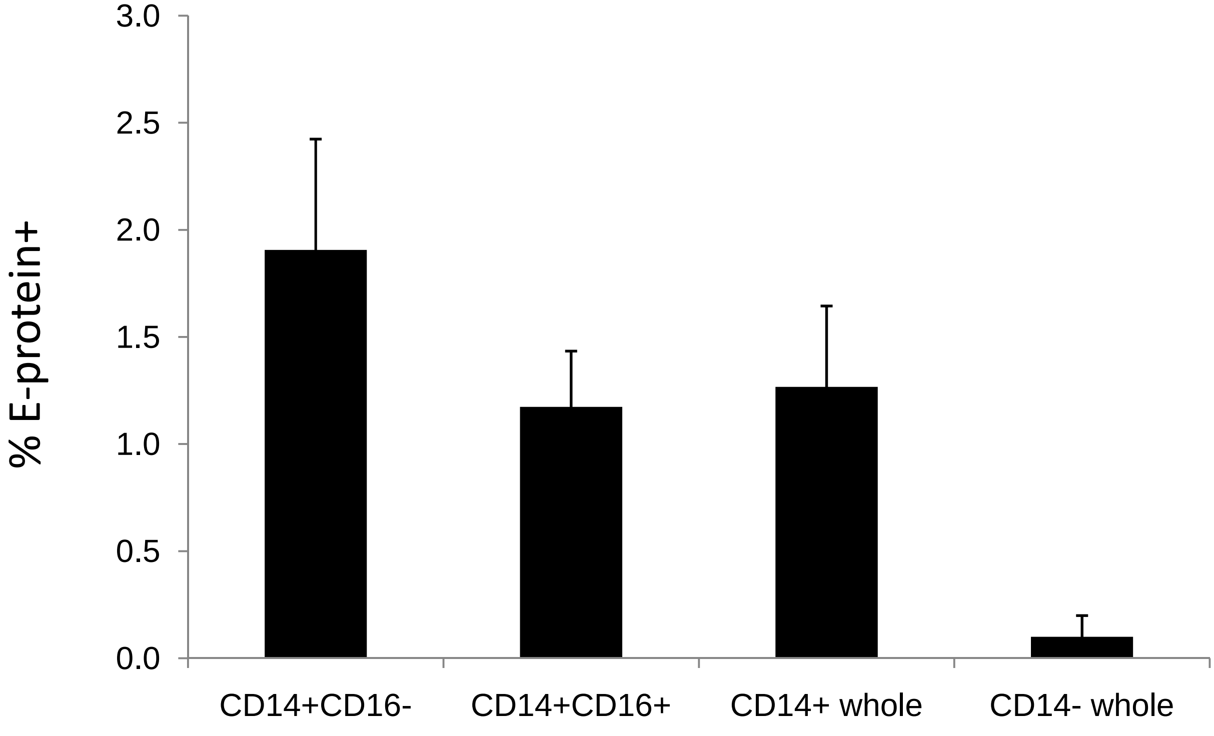

Supplement: Figure S1 — Infection of whole PBMCs. Whole 1 PBMCs were were exposed to dengue virus or medium without virus. After two days, surface staining of CD14 and CD16, and intracellular staining for NS1 and 4G2 was performed. (A) NS1 and 4G2 expression by gating on the whole CD14+ monocyte population. (B) NS1 and 4G2 expression by gating on the whole CD14− non monocyte population. (C) CD14 and CD16 profiles demonstrating the spontaneous upregulation of CD16 by monocytes after two days of culture. (D) NS1 and 4G2 expression by gating on the CD14+CD16+ monocyte population (E) NS1 and 4G2 expression by gating on the CD14+CD16 monocyte population. (F) Percentage of NS1+ and 4G2 positive cells from the mean + SD of three donors. (PDF) [file pone.0036435.s001.pdf]
